# Enterprise digital transformation and customer concentration: An examination based on dynamic capability theory

**DOI:** 10.3389/fpsyg.2022.987268

**Published:** 2022-09-15

**Authors:** Laihui Liu, Suxia An, Xiangyu Liu

**Affiliations:** ^1^School of International Trade and Economics, Shandong University of Finance and Economics, Jinan, China; ^2^School of Accountancy, Shandong University of Finance and Economics, Jinan, China; ^3^College of Economics and Management, Xinjiang Agricultural University, Ürümqi, China

**Keywords:** digital transformation, customer concentration, innovation capability, operating costs, customer satisfaction, dynamic capability theory

## Abstract

Digital transformation of traditional enterprises can better develop new customer relationships and help mitigate the business risk of their over-reliance on single-customer relationships. However, little research has been conducted on the internal mechanisms of how enterprise digitalization reshapes corporate customer relationships. In this manuscript, from the perspective of dynamic capability theory, we construct conceptual models of enterprise digital transformation, innovation capability, operational cost, and customer satisfaction, and explore the internal mechanisms of enterprise digital transformation to reduce the dependence of enterprises on large customers. The model is empirically studied by obtaining data on the degree of digital transformation of enterprises through “search statistics” of keywords in the annual reports of Chinese listed companies during 2011–2019. This manuscript finds that digital transformation significantly reduces the concentration of large customers and has become a powerful driver of business model innovation in the digital economy, and this finding remains robust to the use of PSM and instrumental variable methods to address endogeneity. Digital transformation reduces firms’ dependence on large customers through three mechanisms: improving firms’ innovation capabilities, reducing firms’ operating costs, and increasing customer satisfaction. The impact of digital transformation on reducing the dependence of non-state enterprises on large customers is greater than that of state-owned enterprises; the implementation of digital transformation strategies is more helpful for enterprises that have active interactions with customers to reduce their customer concentration; and the reduction of customer concentration is greater for enterprises in regions with higher levels of digital development compared to those in regions with lower levels of digital development. The economic consequence test finds that digital transformation diversifies customer structure and reduces business risks. The analysis of the innovation effect and customer satisfaction effect on reducing the concentration of large customers of enterprisesby implementing digital transformation enriches and expands the dynamic capability theory and provides important insights for enterprises to diversify their customer structure.

## Introduction

Along with the advent of the fourth industrial revolution, new information technologies such as big data, artificial intelligence, Internet, cloud computing, 5G, etc., have been deeply integrated with the real economy, and data has become a new factor of production driving economic growth in addition to land, capital, labor and technology. At the same time, China’s economic restructuring and industrial transformation and upgrading are facing problems such as rising costs of production factors, aging population, and resource and environmental constraints. The digital economy has become an effective way to effectively address existing challenges and drive a new round of economic growth to achieve a curve. The Chinese government also attaches great importance to the application of digital technology, and by the end of 2020, China’s digital economy will reach 39.2 trillion yuan, accounting for 38.6% of GDP. It has become an important driving force to effectively support epidemic prevention and control and high-quality economic development. Under the influence of the digital economy wave, digital technologies and industrial Internet have accelerated their landing and deepening in key fields such as electronic equipment manufacturing, engineering machinery, electricity, and steel, releasing a multiplier effect to promote the transformation and upgrading of traditional industries. Hence, digital transformation has become a strategic imperative for more and more enterprises (Verhoef et al., 2021). However, traditional enterprises will face unprecedented challenges and obstacles when seeking and implementing digital transformation and business model innovation (Knudsen et al., 2021). Therefore, overcoming digital technology challenges and implementing digital transformation is the key to whether enterprises can break through the cognitive limitations of traditional enterprises and improve their innovation capabilities (Venkataraman et al., 2017).

Since Andal-Ancion et al. (2003) introduced digital transformation into business administration, more and more scholars have conducted research around this topic as digitalization and the real economy have become increasingly integrated (Hess et al., 2016; Venkataraman et al., 2017; Bresciani et al., 2018; Matarazzo et al., 2021). They mainly explored the drivers, underlying mechanisms, and economic and value effects of digital transformation (Karimi and Walter, 2015; Constantinides et al., 2018; Ferreira et al., 2019; Verhoef et al., 2021). Some of these studies are mainly based on the theory of digital empowerment theory (Yoo et al., 2012), relational theory (Sandberg et al., 2020; Siachou et al., 2021), dynamic capability theory (Teece et al., 1997; Soluk and Kammerlander, 2021). However, we should focus more on how the digital transformation of enterprises affects the value reshaping of supply chain relationships (Bresciani et al., 2018; Matarazzo et al., 2021). Customer concentration, an important dimension for measuring business-customer relationships (Patatoukas, 2012; Dhaliwal et al., 2020; Leung and Sun, 2021; Wang et al., 2021), not only affects corporate production and operation decisions (Patatoukas, 2012; Guo et al., 2020), but also has an impact on investors’ investment decisions in the capital market (Luo and Nagarajan, 2015; Cheng et al., 2020).

**FIGURE 1:**
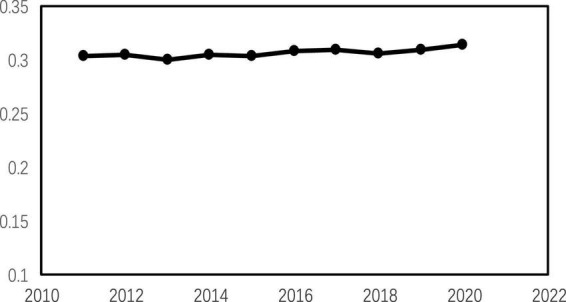
Sales ratio of major customers.

It is well known that customer groups are in the downstream production and sales chain of a company. As a key link in the process of selling a company’s products or services, key customers play an important role in shaping the operations of a company (Patatoukas, 2012; Kim and Henderson, 2015). China has been a relationship-based society since ancient times, and the trade ties between companies and their key customers in the supply chain based on “relationships” are unique to China due to the influence of Confucianism. The average ratio of sales from the top five customers of Chinese listed companies is above 30% (see Figure 1), and the heavy dependence on customers is a distinctive feature of Chinese listed companies. Unlike the positive effects of customer concentration in other countries (Patatoukas, 2012; Ak and Patatoukas, 2016), both theory and practice based on the realities in China confirm that heavy dependence on large customers raises a series of potential risks (Cohen and Li, 2020; Dhaliwal et al., 2020; Guo et al., 2020; Wang et al., 2021). In view of this, the China Securities Regulatory Commission (CSRC) focuses on the customer concentration of companies in its IPO audits of proposed listed companies, listing excessive customer concentration as its risk matter. Therefore, how to reduce the dependence on large customers and optimize the customer structure to promote the sustainable development of enterprises has become a core issue that enterprises urgently need to address at this stage. Enterprises need to establish a scientific thinking of customer relationship management and clarify the process of “digital transformation - enterprise innovation - customer concentration” to better optimize customer relationship management and productivity improvement.

Recent research on digitalization and customer relationships focused on the use of cognitive decision algorithms and data-driven machine learning tools in retail operations to predict customer preferences, brand perceptions, and customer purchase intentions (Hopkins, 2022; Nica et al., 2022) For example, Nica et al. (2022) predicted customer purchase intentions based on neural network algorithms in artificial intelligence technology, and Kliestik et al. (2022) confirmed that shopping with artificial intelligence assistants could shape customer behaviors. As for customer concentration, recent literature focused on how customer concentration affects corporate disclosure decisions (Cheng et al., 2020), and the relationship between economic policy uncertainty and corporate customer concentration (Leung and Sun, 2021). However, existing studies tend to ignore the impact of enterprises’ digital transformation on their customer concentration.

Dynamic capability theory provides a better theoretical basis for this manuscript to analyze the relationship between digital transformation and customer concentration. This theory refers to the formation of new capabilities that can quickly adapt to changes in the external environment by integrating internal and external resources (Teece et al., 1997; Soluk and Kammerlander, 2021). With the advent of the digital era, the external environment faced by enterprises has become more complex and changeable, impeding the production and selling of enterprises. According to the dynamic capability theory, digital transformation can help enterprises perceive complex and changing external market demands and integrate market resources for innovation in time, so as to develop new customers and reduce the concentration of customers. Based on this, this manuscript focuses on the impact of digital transformation on corporate customer concentration and the underlying mechanism, and examines the impact of the unique nature of property rights in China on the relationship between the two. The results find that digital transformation of traditional enterprises helps reduce the concentration of corporate customers and diversify the corporate customer base. This impact is more pronounced in non-state enterprises and those having active interactions with customers and characterized by a higher level of digital development. Mechanistic analysis shows that the relationship between innovation capacity, operating costs and customer satisfaction mediates the relationship between the two. Finally, the finding at the corporate performance level reveals that digital transformation promotes customer structure diversification to enhance corporate performance. Therefore, this study is of great relevance in promoting the sustainable development of enterprises.

This study contributes to dynamic capability theory and the research about enterprise-customer relationship in the following ways. First, this manuscript extends the existing research perspective on customer concentration by showing that digital transformation can reshape the enterprise-customer relationship. Dynamic capability theory is integrated with corporate digital transformation to introduce the mechanisms underlying the impact of digital transformation on customer concentration. Thus, this study reveals, to a certain extent, the positive role of digitally empowered enterprise transformation in reshaping enterprise-customer relationships. Second, the empirical evidence in this manuscript suggests that enterprises with higher levels of digital transformation are more likely to help drive customer structure diversification and reduce corporate customer concentration. Although recent research has explored the impact of digitalization (intelligence) on customer behavior choices, it was largely limited to the retail operations of individual enterprises, neglecting the exploration in cross-organizational relationships. To my knowledge, this manuscript is one of the first attempts to examine the impact of corporate digital transformation on customer relationships in a cross-firm context. Third, this study focuses on the moderating role of the nature of corporate ownership in the relationship between digital transformation and customer concentration, confirming that the role of digital transformation is influenced by the differential nature of corporate ownership. It enriches the theory of dynamic capabilities in the context of a digital boom.

## Theoretical analysis and research hypotheses

In recent years, against the intensified trade friction between China and the U.S. and the impact of the COVID-19, which increased the uncertainty in international market and trade relationships between companies and overseas customers, more and more companies are actively exploring their potential domestic customer base. The digital economy era provides opportunities for digital transformation of enterprises. Digital transformation fuels the shift of traditional businesses to end-to-end services, mass customization, and customer centricity (Weichert, 2017), resulting in an increasing blurring of the boundaries between the physical economy and the online industrial structure and further bridging the physical economy and online content to create an omnichannel environment for customers (Brynjolfsson et al., 2013). In addition, although redeveloping new customer relationships faces higher costs and requires significant marketing, investment, and production capacity, diversifying the customer mix can provide greater benefits to firms, given the increasing demand for personalization by customers (Leung et al., 2021). Portfolio theory suggests that diversification of revenue sources reduces the overall risk of a firm (Markowitz, 1952). Therefore, digital transformation helps enterprises diversify their customer portfolios and reduce the risk of single-customer dependence.

Dynamic capabilities theory provides a very fitting perspective for analyzing the potential mechanisms by which digital transformation affects customer concentration. Dynamic capabilities of enterprises include perceptive capabilities, acquisition capabilities, and reconfiguration capabilities (Teece, 2018; Soluk and Kammerlander, 2021). According to this theory, companies undergoing digital transformation respond to changes in the external environment by enhancing their own innovation capabilities, integrate internal resources to effectively reduce corporate operating costs, and reconfigure their business models to improve customer satisfaction, thereby reducing customer concentration and promoting customer structure diversification. This manuscript further discusses how digital transformation can shape dynamic capabilities in three aspects: innovation capability, operation cost, and customer satisfaction, and thus reduce customer concentration.

(1) Digital transformation improves the ability of companies to innovate, which in turn reduces the concentration of large customers. Firstly, organizational learning theory suggests that integrating new information is the basis for organizations to improve their innovation capabilities (Hyysalo et al., 2017), and digital transformation brings incremental innovation to firms by processing much data about customer behavior to better understand customers. The application of next-generation information technology helps firms process the data about customer needs (for example, what to buy and where to buy) and provides them with a more complete and comprehensive market perception (Kamakura and Du, 2012), thus improving their ability to understand customer needs and exploiting untapped opportunities. This knowledge acquisition process helps firms identify products that can meet future market needs, improve their innovation capabilities (Erevelles et al., 2016), diversify their customers, and reduce their dependence on major customers. Secondly, the deep integration of digital and entity enterprises helps promote a new model of networked collaborative innovation (Rodriguez-Lluesma et al., 2021). The new model can realize the collaboration between enterprise innovation and design and downstream customers in the supply chain, accelerate the exchange and sharing of data on industrial collaborative innovation, improve the probability of successful innovation, provide timely feedback on the needs of downstream customers in the supply chain, accelerate enterprises’ upgrading and optimization of products and services, and enhance their market competitiveness. As a result, with increased R&D investment and innovation capabilities, companies can produce more diverse and differentiated new products, acquire new customers more easily, and redistribute revenues among different customers because they have the ability to meet the specific needs of new customers.

(2) Digital transformation can reduce the cost of business operations and customer concentration. Traditional customer relationships prefer to be stable (Patatoukas, 2012) because firms believe that major customers reduce the cost of expanding new customers and provide stable cash inflows for firms. However, existing studies confirm that the disadvantages of high customer concentration outweigh the benefits (Cohen and Li, 2020; Wang et al., 2021). Higher customer concentration indicates stronger bargaining power of major customers (Gosman and Kohlbeck, 2009). They cannot only shift operational risk by depressing corporate prices and adjusting order sizes, but also lead to significant losses of corporate investments in dedicated assets due to unexpected termination of contracts (Guo et al., 2020). In contrast, firms engage in digital transformation strategies to enhance their market competitiveness by reducing corporate costs to gain first mover advantage (Matt et al., 2015), enhance their bargaining power over customers, and thus consolidate and expand customer resources. Firstly, digital transformation can boost the construction of information technology, promote the intelligent transformation of enterprises through the use of artificial intelligence, machine learning and other technologies, accelerate the data acquisition and storage, improve the data agility (Weichert, 2017), and reduce the cost of information collection. Secondly, digital transformation can facilitate the construction of corporate digital platforms, which reduces the cost of business operations. New marketing channels can be easily accessed by smartphones and tablets (Parker et al., 2016), thus bringing a revolutionary way of implementing corporate brand communication strategies (Lamberton and Rose, 2012), stimulating an ongoing dialog with customers, influencing the meaning and message of brands, governing the development of products or services, and increasing customer base diversity.

(3) Digital transformation improves customer satisfaction and thus reduces the dependence of companies on major customers. Firstly, digital transformation can extend the boundaries of a company’s business and services (Kohtamäki et al., 2020) and meet the differentiated and personalized needs of customers. The application of digital technologies such as the Internet, artificial intelligence and apps transforms companies from merely offering products to providing targeted products and after-sales services (Visnjic et al., 2018). This not only improves the experience of customers but also expands the added value of companies from product production only to multiple product-related services (Huikkola and Kohtamäki, 2018), increasing customer satisfaction. Secondly, digital transformation helps shift the business model from the traditional “product-centric” model to “customer-centric” model (Kohtamäki et al., 2020). Companies match customer needs precisely with products through digital products, digital technologies, and the construction of digital platforms, and provide solutions to meet customer needs. They continue to expand their services such as information consulting, operational services, and product innovation to improve customer satisfaction. Finally, digital transformation can create a complex digital ecosystem (Knudsen et al., 2021). In this ecosystem, by integrating advantageous resources, forming a networked, industry-wide ecosystem, sharing, utilizing, and recreating knowledge, data, and heterogeneous resources, and then opening up service flows such as organizational management, product design, production and operations, logistics, and social networks, companies can improve their perceptual capabilities in deep connection with customers in order to enhance customer experience, thus improving customer satisfaction. Based on this, the following hypotheses are proposed.

H1: Digital transformation reduces customer concentration and helps companies diversify their customers.

H2: Digital transformation reduces customer concentration by improving companies’ innovation capabilities.

H3: Digital transformation reduces customer concentration by reducing companies’ costs.

H4: Digital transformation reduces customer concentration by improving customer satisfaction.

## Materials and methods

### Sample selection and data sources

This manuscript selects listed companies from A-share markets in Shanghai and Shenzhen between 2011 and 2019 as the initial research samples, and the data sample period starts from 2011 mainly because the rapid expansion of the Internet and the rapid development of digital economy in China started after 2010. The data from annual reports used to calculate the indicators of digital transformation in this manuscript are taken from the Cninfo website. The data about customers and social, environmental and governance aspects of enterprises are extracted from CNRDS database. The financial data of enterprises are selected from CSMAR database. In order to ensure the credibility of the research, by using the approaches of Kohtamäki et al. (2020) and Matt et al. (2015), the sample screening was conducted as following: (1) excluding the listed companies in finance or insurance-related industries; (2) excluding the ST and *ST listed companies and the delisted ones; (3) excluding listed companies in the year of IPO; (4) excluding the listed companies with incomplete or missing financial index data. After the above steps, a total of 11,560 companies are selected.

### Regression model

Considering the studies of Kohtamäki et al. (2020) and Leung et al. (2021), the impact of digital transformation of firms on customer concentration is examined by constructing model (1).


(1) $$\begin{array}{c} C\,u\,s\,t\,o\,m\,e\,r_{i,t}=\eta_{0}+\eta_{1}\,D\,I\,G\,I_{i,t-1}+\eta_{2}\,S\,i\,z\,e_{i,t}+\eta_{3}\,L\,e\,v_{i,t}+\\ \eta_{4}\,R\,o\,a_{i,t}+\eta_{5}\,C\,a\,s\,h_{i,t}+\eta_{6}\,A\,t\,u\,r\,n_{i,t}+\eta_{7}\,T\,o\,p\,1_{i,t}+\\ \eta_{8}\,D\,u\,a\,l_{i,t}+\eta_{9}\,I\,n\,d\,e\,p_{i,t}+\eta_{10}\,S\,o\,e_{i,t}+\eta_{11}\,A\,G\,D\,P_{i,t}\\ +\eta_{12}\,M\,a\,r\,k\,e\,t_{i,t}+\eta_{13}\,I\,n\,t\,e\,r\,n\,e\,t_{i,t}+\sum Y\,e\,a\,r+\\ \sum I\,n\,d\,u\,s\,t\,r\,y+\sum C\,i\,t\,y+\xi_{i,t} \end{array}$$

Where Customer represents customer concentration; DIGI is digital transformation of enterprises; η_0_ is the intercept term, and η_1_ is the regression coefficient of the explanatory variables. If the regression coefficient η_1_ is significantly negative, it indicates that the higher degree of digital transformation of enterprises means the lower customer concentration of enterprises. η_2_–η_13_ are the regression coefficients of control variables, and ζ is random error terms. The model also controls the fixed effects of industry, year and city to avoid the interference of relevant factors such as unobserved industry characteristics and city-level characteristics on the regression results of this manuscript.

### Variable selection and measurement

#### Explanatory variable: Customer concentration (customer)

considering the studies of Patatoukas (2012) and Dhaliwal et al. (2016), customer concentration (Customer) is measured by (1) the share of sales revenue from the top five customers in the current year’s sales revenue (Cus_Top5) and (2) the Herfindahl index of sales revenue from the top five customers. The latter index (Cus_HHI) is calculated as follows:


(2) Cus_HHIi⁢t=∑j=1J(Sales/i⁢j⁢tSales)i⁢t2

Where *Sales*_*ijt*_ refers to company *i*’s sales revenue from customer *j* in year *t*, and *Sales*_*it*_ refers to company *i*’s sales revenue from its top five customers in year t. Higher Cus_HHI indicates higher customer concentration.

#### Explanatory variable: Digital transformation index

In this manuscript, Python is used to download the annual reports of listed companies from the Cninfo website, and Java PDFbox is adopted to extract the text content of “Management Discussion and Analysis (MD&A)” in the annual reports. Thus, a lexicon about digital transformation is constructed (see Table 1). The key words about digital transformation in MD&A are identified and their frequency is counted. Then, the frequency of all the words based on logarithmic processing is summarized to obtain the digital transformation index (DIGI).

**TABLE 1:** Word frequency mapping of digital transformation.

Internet	Mobile internet, industrial internet, mobile internet, internet healthcare, e-commerce, mobile payment, third-party payment, NFC payment, smart energy, online, offline, B2B, B2C, C2B, C2C, O2O, netlink, smart wear, smart agriculture, smart transportation, smart healthcare, smart customer service, smart home, smart investment, smart culture and tourism, smart environmental protection, smart Electricity network, intelligent marketing, digital marketing, unmanned retail, Internet finance, digital finance, fintech, financial technology, quantitative finance, and open banking
Big data	Big data, data mining, text mining, data visualization, heterogeneous data, credit, augmented reality, mixed reality, and virtual reality
Cloud computing	Cloud computing, streaming computing, graph computing, in-memory computing, multi-party secure computing, green computing, information physical systems, brain-like computing, Internet of Things, cognitive computing, converged architectures, billion-level concurrency, and EB-level storage
Artificial intelligence	Artificial intelligence, biometrics, face recognition, business functions, intelligent data, analytical image understanding, investment decision aids, intelligent robotics, machine learning, deep learning, semantic search, voice recognition, identity verification, natural language processing, and autonomous driving
Blockchain	Blockchain, digital currency, distributed computing, differential privacy technology, and smart financial contracts

#### Control variables

Referring to the studies of Kohtamäki et al. (2020) and Leung et al. (2021), this manuscript considers the variables such as firm size (Size), asset liability ratio (Lev), return on assets (Roa), cash flow intensity (Cash), total asset turnover ratio (Aturn), shareholding ratio of the largest shareholder (Top 1), two positions in one (Dual), and the percentage of independent directors (Indep). The regional-level control variables include the level of per capita GDP (AGDP), the degree of marketization (Market), and the Internet penetration (Internet) in the region where the firm is located for control. In addition to the above control variables, the industry-, year-, and city-level fixed effects are controlled in the regression model, and the standard errors are adjusted for heteroskedasticity. The specific variable definitions are detailed in Table 2.

**TABLE 2:** Variable definitions and descriptions.

Name variable	Symbol variable definition
Cus_Top5	Sales revenue from top five customers as a percentage of sales revenue for the year
*Cus_HHI*	Herfindahl index of sales revenue from top five customers
*DIGI*	See variable definitions for details
Size	Natural logarithm of total corporate assets
*Lev*	Total corporate liabilities/total assets
Roa	Net cash flows from operating activities of enterprises/total assets
*Cash*	(Cash and cash equivalents)/total assets
*Aturn*	Net operating income/average total assets
*Top1*	The ratio of the majority shareholder’s shareholding to the total number of shares in year t
*Dual*	Dual is a dummy variable, 1 if the CEO and chairman of the firm are the same person, 0 otherwise
*Indep*	Number of independent directors/number of board of directors
*Loss*	dummy variable, 1 if the firm had a loss in the previous year, 0 otherwise
*AGDP*	Natural logarithm of GDP per capita in the region
*Market*	Marketability index of the province where the company is located (2019 version)
*Year*	Annual dummy variables
*Industry*	Industry dummy variables
*City*	City dummy variables

## Results and analysis

### Descriptive statistics

To eliminate the possible influence of extreme values, financial variables were winsorized according to the 1% quantile in this manuscript. The descriptive statistics of main variables in Table 3 show that, from the indicators related to major customers, the mean value of customer concentration is 0.305 and the standard deviation is 0.218, indicating that there are significant differences in the concentration of customers between samples. In terms of digital transformation, the mean value is 2.827, and standard deviation is 1.518, revealing significant differences in the degree of digital transformation of samples. The mean values of firm size, asset liability ratio, return on assets, cash holding level, total asset turnover ratio, and shareholding ratio of the largest shareholder are 22.153, 42.2, 4.4, 15.8, 61.7, and 34.251%, respectively. Nearly 27% of the samples have two positions of chairman and general manager in one, indicating that there are significant differences among companies in terms of corporate finance and governance. The simple correlation coefficients between the main variables in Table 2 show that DIGI has a significant negative correlation with Cus_Top5 and Cus_HHI. It indicates that digital transformation reduces corporate customer concentration, which is in line with the expectation of the hypothesis H1. Given that the correlation analysis only reflects the simple correlation between the variables, further regression analysis is needed to verify the test of hypothesis H1. Meanwhile, except for the high correlation between Cus_Top5 and Cus_HHI, the correlation coefficients between the other variables are low and basically below 0.5, indicating that there is no serious multicollinearity between the variables.

**TABLE 3:** Descriptive statistics and correlation coefficient matrix.

	Mean	Standard deviation	1	2	3	4	5	6
*1. Cus_Top5*	0.305	0.218	1					
*2. Cus_HHI*	0.055	0.116	0.785***	1				
*3. DIGI*	2.827	1.518	−0.030**	−0.069***	1			
*4. Size*	22.153	1.251	−0.082***	0.066***	−0.082***	1		
*5. Lev*	0.422	0.205	−0.054***	0.046***	−0.163***	0.415***	1	
*6. Roa*	0.044	0.074	−0.038***	0.019	−0.096***	0.139***	−0.028**	1
*7. Cash*	0.158	0.122	−0.027**	−0.061***	0.113***	−0.139***	−0.251***	–0.005
*8. Aturn*	0.617	0.429	−0.140***	−0.094***	–0.016	0.070***	0.163***	0.089***
*9. Top1*	34.251	14.906	0.043***	0.106***	−0.158***	0.275***	0.123***	0.133***
*10. Indep*	3.171	0.578	–0.003	0.071***	−0.097***	0.330***	0.195***	0.069***
*11. Dual*	0.270	0.444	−0.027**	−0.051***	0.118***	−0.074***	0.0150	−0.140***
*12. Soe*	0.329	0.470	0.057***	0.137***	−0.253***	0.342***	0.274***	0.065***
*13. Market*	10.857	2.353	−0.031***	−0.067***	0.242***	−0.061***	−0.112***	−0.024**
*14. AGDP*	10.086	3.600	–0.004	0.003	−0.056***	−0.038***	−0.015*	0.021***

	**7**	**8**	**9**	**10**	**11**	**12**	**13**	**14**

*7. Cash*	1							
*8. Aturn*	0.003	1						
*9. Top1*	0.005	0.119***	1					
*10. Indep*	–0.019	0.021*	0.089***	1				
*11. Dual*	−0.103***	−0.055***	−0.057***	−0.101***	1			
*12. Soe*	0.027**	0.116***	0.272***	0.252***	−0.191***	1		
*13. Market*	–0.001	0.002	−0.047***	−0.125***	0.083***	−0.202***	1	
*14. AGDP*	–0.010	−0.021*	0.084***	0.050***	−0.122***	0.100***	0.034***	1

Pearson coefficients in the lower left; *p < 0.1, **p < 0.05, ***p < 0.01.

### Baseline results

Table 4 reports the results of the baseline regressions of model (2) with the explanatory variables Cus_Top5 and Cus_HHI for corporate customer concentration (L.DIGI). Columns (1) and (2) are the results of univariate regressions, and columns (3) and (4) refer to the results of regressions after adding control variables. All regressions in the table control the fixed effects at the levels of year, industry and city. The results in columns (1) and (2) show that corporate digital transformation (L.DIGI) has a significantly negative correlation with Cus_Top5 and Cus_HHI at the 1% level, and after adding the control variables, corporate digital transformation (L.DIGI) has a significantly negative correlation with Cus_Top5 and Cus_HHI at the 1% level, supporting the hypothesis H1 that corporate digital transformation significantly reduce customer concentration.

**TABLE 4:** Enterprise digital transformation and customer concentration.

	(1)	(2)	(3)	(4)
	
	*Cus_Top5*	*Cus_HHI*	*Cus_Top5*	*Cus_HHI*
*L.DIGI*	−0.0091***	−0.0033***	−0.0086***	−0.0029***
	(−5.4771)	(−4.0220)	(−5.1529)	(−3.4089)
*Size*			−0.0324***	−0.0068***
			(−15.1679)	(−5.5838)
*Lev*			0.0289**	0.0138*
			(2.1083)	(1.8230)
*Roa*			−0.0877***	–0.0009
			(−3.1625)	(−0.0596)
*Cash*			0.0217	0.0080
			(1.1224)	(0.8161)
*Aturn*			−0.0410***	−0.0074**
			(−7.5865)	(−2.5569)
*Top1*			0.0005***	0.0004***
			(3.3397)	(5.4876)
*Indep*			–0.0047	0.0004
			(−1.2516)	(0.1857)
*Dual*			−0.0085**	−0.0040**
			(−2.0290)	(−2.1291)
*Soe*			0.0137***	0.0021
			(2.7161)	(0.8538)
*Market*			0.0078	0.0058
			(1.0165)	(1.3671)
*AGDP*			−0.0019**	–0.0006
			(−2.0286)	(−1.4331)
*Year*	*Yes*	*Yes*	*Yes*	*Yes*
*Industry*	*Yes*	*Yes*	*Yes*	*Yes*
*City*	*Yes*	*Yes*	*Yes*	*Yes*
_cons	0.4731***	0.0745***	1.1627***	0.1730***
	(16.6702)	(4.8928)	(16.1964)	(4.3242)
adj. *R*^2^	0.257	0.299	0.287	0.304
N	11, 560	11, 560	11, 560	11, 560

*P < 0.1, **P < 0.05, ***P < 0.01. The same below.

### Robustness checks

#### Endogeneity

First, although the firm-level and region-level influences are controlled in the main test, there may be still some unobservable factors that can affect the regression results. For this reason, this manuscript uses propensity score matching (PSM) to overcome the endogeneity problem that may arise due to omitted variables. In this manuscript, firms that underwent digital transformation are defined as the experimental group, and those that did not undergo it are defined as the control group. The regressions are conducted after one-to-one nearest neighbor matching, kernel matching, and Mahalanobis matching, with the control variables in the main regression as covariates. The regression results in columns (1)–(6) in Table 5 show that corporate digital transformation (L.DIGI) and customer concentration (Cus_Top5 and Cus_HHI) are both significant at the 1% level, revealing that the findings of this manuscript are still robust and reliable after excluding the problem of omitted variables due to model setting bias.

**TABLE 5:** PSM and instrumental variables test results.

	(1)	(2)	(3)	(4)	(5)	(6)	(7)	(8)	(9)

	Nearest neighbor matching		Kernel matching		Marksmanship matching		Tool variable test		
				
	*Cus_Top5*	*Cus_HHI*	*Cus_Top5*	*Cus_HHI*	*Cus_Top5*	*Cus_HHI*	*DIGI*	*Cus_Top5*	*Cus_HHI*
*L.DIGI*	−0.0091***	−0.0031***	−0.0089***	−0.0030***	−0.0104***	−0.0031***		−0.0049*	−0.0036**
	(−4.8450)	(−2.9454)	(−4.7854)	(−2.9131)	(−5.0667)	(−2.6873)		(−1.8801)	(−2.5622)
*DIGI_ind*							0.3022***		
							(3.8779)		
*Size*	−0.0316***	−0.0069***	−0.0306***	−0.0069***	−0.0352***	−0.0080***	0.1003***	−0.0265***	−0.0026**
	(−13.6866)	(−5.3519)	(−13.3483)	(−5.3442)	(−12.8933)	(−5.1768)	(8.4117)	(−12.6797)	(−2.2897)
*Lev*	0.0085	0.0092	0.0083	0.0087	0.0454***	0.0293***	−0.5126***	0.0159	0.0138*
	(0.5671)	(1.1110)	(0.5571)	(1.0477)	(2.6583)	(3.0371)	(−6.6816)	(1.2099)	(1.9488)
*Roa*	−0.0729**	0.0028	−0.0766**	–0.0015	−0.0798**	0.0210	–0.1689	−0.0988***	0.0126
	(−2.2990)	(0.1573)	(−2.4725)	(−0.0857)	(−2.0125)	(0.9381)	(−1.0615)	(−3.3759)	(0.8027)
*Cash*	0.0229	0.0001	0.0165	0.0037	0.0489**	0.0108	0.7650***	−0.0841***	−0.0298***
	(0.8857)	(0.0047)	(0.6565)	(0.2601)	(1.9775)	(0.7694)	(7.4277)	(−4.5023)	(−2.9751)
*Aturn*	−0.0325***	−0.0074**	−0.0348***	−0.0076**	−0.0404***	−0.0101***	–0.0245	−0.0834***	−0.0327***
	(−5.3533)	(−2.1992)	(−5.7558)	(−2.2411)	(−5.8888)	(−2.5952)	(−0.7859)	(−17.4338)	(−12.7270)
*Top1*	0.0005***	0.0005***	0.0005***	0.0005***	0.0005***	0.0005***	−0.0030***	0.0010***	0.0006***
	(3.2504)	(5.6774)	(3.1269)	(5.1354)	(2.8284)	(4.6901)	(−3.8527)	(6.8252)	(7.6901)
*Indep*	–0.0027	0.0014	–0.0043	0.0012	–0.0034	0.0016	0.0049	0.0006	0.0033*
	(−0.6858)	(0.6490)	(−1.0725)	(0.5489)	(−0.7590)	(0.6316)	(0.2433)	(0.1694)	(1.6841)
*Dual*	–0.0050	–0.0031	–0.0055	–0.0024	–0.0039	–0.0042	0.1604***	−0.0118**	−0.0047*
	(−0.8360)	(−0.9324)	(−0.9224)	(−0.7304)	(−0.6305)	(−1.1958)	(6.3293)	(−2.5014)	(−1.8650)
*Soe*	0.0094*	0.0013	0.0081	0.0010	0.0164***	0.0035	−0.3727***	0.0428***	0.0232***
	(1.6870)	(0.4033)	(1.4480)	(0.3097)	(2.6089)	(0.9843)	(−12.8735)	(8.3844)	(8.4743)
*Market*	–0.0020	0.0025	–0.0027	0.0018	0.0031	0.0028	–0.0098	–0.0011	−0.0010**
	(−0.2246)	(0.5173)	(−0.3053)	(0.3612)	(0.3457)	(0.5373)	(−0.2267)	(−1.2266)	(−2.0954)
*AGDP*	–0.0015	–0.0003	–0.0017	–0.0003	–0.0025	–0.0006	–0.0011	–0.0005	–0.0003
	(−1.4624)	(−0.5225)	(−1.6135)	(−0.5681)	(−1.5240)	(−0.5951)	(−0.1806)	(−0.8500)	(−0.8800)
*Year*	Yes	Yes	Yes	Yes	Yes	Yes	Yes	Yes	Yes
*Industry*	Yes	Yes	Yes	Yes	Yes	Yes	Yes	Yes	Yes
*City*	Yes	Yes	Yes	Yes	Yes	Yes	Yes	Yes	Yes
_cons	1.1913***	0.1846***	1.1843***	0.1917***	1.2459***	0.2100***	−1.8329***	0.9130***	0.1181***
	(11.8007)	(3.2831)	(11.7522)	(3.3903)	(10.8857)	(3.2442)	(−3.3013)	(20.9709)	(5.0515)
adj. *R*^2^	0.2881	0.3032	0.2917	0.3071	0.2790	0.2947	0.4696	0.0463	0.0363
*N*	8275	8275	8430	8430	6961	6961	11559	11559	11559

Second, the empirical study in this manuscript may have endogenous endosomes due to reverse causality, i.e., the higher dependence on customers means the greater inclination to undergo digital transformation, making it possible that the decrease in customer concentration may not be due to digital transformation. Based on the studies of Kohtamäki et al. (2020), two-stage least squares estimation was conducted using the mean of the digital transformation index of the industry in which the firm operates as an instrumental variable. The regression results are detailed in columns (7)–(9) of Table 5. The regression coefficient for digital transformation (L.DIGI) is significantly negative, consistent with the main regression results. This indicates that the regression results remain robust after controlling endogeneity issues.

### Alternative measures for the key variables

(1) Replacement of the explanatory variable. After replacing the explanatory variable with the share of sales of the largest customer (Cus_top1), the regression results in columns (1) and (2) show that the regression coefficients of digital transformation (L.DIGI) are both significantly negative at the 1% level. Constructing whether the firm undergoes digital transformation (L.DIGI_dum) replaces the explanatory variable, the regression results in columns (3) and (4) of Table 6 show that the regression coefficients of digital transformation (L.DIGI_dum) are both significantly negative at the 1% level. This indicates that the results of the main regression remain robust after the redefinition of main variables.

**TABLE 6:** Robustness test.

	(1)	(2)	(3)	(4)	(5)	(6)
	*Cus_top1*	*Cus_top1*	*Cus_Top5*	*Cus_HHI*	*Cus_Top5*	*Cus_HHI*
L.DIGI	−0.0047***	−0.0042***			−0.0122***	−0.0040***
	(−4.1276)	(−3.7319)			(−6.9474)	(−4.4193)
*L.DIGI_dum*			−0.0186***	−0.0085***		
			(−3.7341)	(−3.2587)		
Size		−0.0124***	−0.0328***	−0.0069***	−0.0351***	−0.0077***
		(−8.7049)	(−16.3326)	(−6.5115)	(−15.8074)	(−5.8692)
Lev		0.0154*	0.0312**	0.0144**	0.0352**	0.0192**
		(1.6861)	(2.4145)	(2.1091)	(2.4452)	(2.3628)
Roa		−0.0395**	−0.0841***	0.0006	−0.0596**	0.0114
		(−2.0879)	(−3.1436)	(0.0450)	(−2.0402)	(0.7238)
Cash		0.0151	0.0179	0.0071	0.0232	0.0086
		(1.2285)	(1.0330)	(0.7742)	(1.1356)	(0.8206)
Aturn		−0.0141***	−0.0412***	−0.0075***	−0.0468***	−0.0098***
		(−3.7834)	(−7.8447)	(−2.7161)	(−8.4280)	(−3.2178)
Top1		0.0005***	0.0005***	0.0004***	0.0006***	0.0005***
		(5.2188)	(3.7523)	(6.1013)	(3.9955)	(5.7575)
Indep		–0.0009	–0.0045	0.0006	–0.0031	0.0012
		(−0.3690)	(−1.3077)	(0.3159)	(−0.7702)	(0.4700)
Dual		–0.0047	−0.0093**	−0.0042*	–0.0031	–0.0026
		(−1.5661)	(−2.1836)	(−1.8549)	(−0.7114)	(−1.3204)
Soe		0.0067*	0.0156***	0.0026	0.0126**	0.0020
		(1.9416)	(3.1926)	(0.9961)	(2.3836)	(0.7623)
Market		0.0054	0.0072	0.0056	0.0056	0.0039
		(1.0461)	(0.9958)	(1.4564)	(0.7088)	(0.8970)
AGDP		−0.0012*	−0.0020**	–0.0007	−0.0022**	–0.0006
		(−1.6773)	(−2.0445)	(−1.2964)	(−2.1922)	(−1.3524)
Year	Yes	Yes	Yes	Yes	Yes	Yes
Industry	Yes	Yes	Yes	Yes	Yes	Yes
City	Yes	Yes	Yes	Yes	Yes	Yes
_cons	0.2208***	0.4556***	1.1749***	0.1770***	1.2256***	0.1973***
	(6.6367)	(6.9663)	(12.7125)	(3.6326)	(16.5325)	(4.7330)
Adj. R^2^	0.2629	0.2714	0.2688	0.2876	0.2861	0.3031
N	11546	11546	11560	11560	10434	10434

(2) Replacement of regression samples. Considering that the computer, communication, and other electronic equipment manufacturing industries have a natural connection with digital technology, in order to reduce their influence on the regression results, the regression results are detailed in columns (5) and (6) of Table 6. The regression coefficients of digital transformation (L.DIGI) are all significantly negative at the 1% level, indicating that the results of the main regression remain robust after the redefinition of main variables.

#### Examination of the mechanism of action

The aforementioned research has provided an overall picture of “corporate digital transformation-customer concentration” and empirically examined the differences in the effects of digital transformation in various aspects, but has not yet clarified its mechanism of action. In the theoretical analysis, this manuscript argues that digital transformation can reduce customer concentration by improving innovation capability, reducing operating costs and increasing customer satisfaction. To test the role of the above three channels, this manuscript constructs model (3) and model (4) with reference to Baron and Kenny’s (1986) mediation effect test procedure to verify the above three transmission paths.


(3) $$\begin{array}{c} M_{i,t}=\alpha_{0}+\alpha_{1}\,D\,I\,G\,I_{i,t-1}+\alpha_{2}\,S\,i\,z\,e_{i,t}+\alpha_{3}\,L\,e\,v_{i,t}+\alpha_{4}\,R\,o\,a_{i,t}+\\ \alpha_{5}\,C\,a\,s\,h_{i,t}+\alpha_{6}\,A\,t\,u\,r\,n_{i,t}+\alpha_{7}\,T\,o\,p\,1_{i,t}+\alpha_{8}\,D\,u\,a\,l_{i,t}+\\ \alpha_{9}\,I\,n\,d\,e\,p_{i,t}+\alpha_{10}\,S\,o\,e_{i,t}+\alpha_{11}\,A\,G\,D\,P_{i,t}+\alpha_{12}\,M\,a\,r\,k\,e\,t_{i,t} \end{array}$$

$$\begin{array}{c} +\alpha_{13}\,I\,n\,t\,e\,r\,n\,e\,t_{i,t}+\sum Y\,e\,a\,r+\sum I\,n\,d\,u\,s\,t\,r\,y\\ +\sum C\,i\,t\,y+\xi_{i,t} \end{array}$$

(4) $$\begin{array}{c} C\,u\,s\,t\,o\,m\,e\,r_{i,t}=\beta_{0}+\beta_{1}\,D\,I\,G\,I_{i,t-1}+\beta_{2}\,M_{i,t}+\beta_{3}\,S\,i\,z\,e_{i,t}+\beta_{4}\,L\,e\,v_{i,t}+\\ \beta_{5}\,R\,o\,a_{i,t}+\beta_{6}\,C\,a\,s\,h_{i,t}+\beta_{7}\,A\,t\,u\,r\,n_{i,t}+\beta_{8}\,T\,o\,p\,1_{i,t}\\ +\beta_{9}\,D\,u\,a\,l_{i,t}+\beta_{10}\,I\,n\,d\,e\,p_{i,t}+\beta_{11}\,S\,o\,e_{i,t}+\\ \beta_{12}\,A\,G\,D\,P_{i,t}+\beta_{13}\,M\,a\,r\,k\,e\,t_{i,t}+\beta_{14}\,I\,n\,t\,e\,r\,n\,e\,t_{i,t}+\\ \sum Y\,e\,a\,r+\sum I\,n\,d\,u\,s\,t\,r\,y+\sum C\,i\,t\,y+\omega_{i,t} \end{array}$$

Where M represents the corporate innovation capability (R&D investment = the ratio of R&D expenditure to main business revenue; innovation output Lnpat = the natural logarithm of patent applications of firms); operating cost (Cost = (main business cost+ administrative expense + selling expense)/main business revenue); customer satisfaction (Sati = relative market share, which is the market share of the firm relative to the largest competitor in the industry share of sales) three mediating variables, model (3) and model (4) of the mediating effect test model, the main focus on the coefficients of α_*1*_ and β_*2*_, if both are significant, it is proved that the mediating effect holds. If β_*1*_ is not significant, this indicates that M is fully mediated, and if β_*1*_ remains significant, this reveals that M is partially mediated.

#### Innovation capability

For promoting corporate digital transformation, more R&D and innovation activities need to be conducted to enhance the internal drive of innovation-driven digital transformation, and innovation capability enhancement will further reduce the dependence of enterprises on major customers. For the mechanism test of innovation capability, corporate R&D investment (RD) and innovation output (Lnpat) are used as mediating variables. From the regression results in Table 7, the regression coefficients of digital transformation (L.DIGI) in column (1) are significantly positive at the 1% level; the results in columns (2) and (3) show that the regression coefficients of corporate R&D investment (RD) are significantly negative at the 5% level; the coefficient of digital transformation (L.DIGI) is significantly negative after controlling R&D investment (RD), indicating that the effect of corporate digital transformation on customer concentration is realized partly through increased corporate innovation investment. Similarly, columns (4)–(6) show the results of the mediating effect test on corporate innovation output, which reveal that corporate digital transformation promotes patent innovation output. The above results indicate that corporate digital transformation can provide more information and interpretation for corporate innovation activities, help enterprises innovate more effectively based on the feedback of customer needs, improve customer satisfaction, reduce customer satisfaction, and improve customer structure diversification.

**TABLE 7:** Test results of mediating effects of firms’ innovation capability.

	(1)	(2)	(3)	(4)	(5)	(6)
	*RD*	*Cus_Top5*	*Cus_HHI*	*Lnpat*	*Cus_Top5*	*Cus_HHI*
*L.DIGI*	0.4261***	−0.0082***	−0.0029***	0.1243***	−0.0081***	−0.0025***
	(11.8561)	(−5.1264)	(−3.4631)	(11.0545)	(−5.1228)	(−2.9629)
RD		−0.0010**	−0.0005**			
		(−2.3804)	(−2.1009)			
*Lnpat*					−0.0035***	−0.0031***
					(−2.6303)	(−4.4498)
*Size*	−0.1178**	−0.0325***	−0.0026**	0.5260***	−0.0306***	−0.0052***
	(−2.5753)	(−16.1621)	(−2.2376)	(36.7548)	(−14.3653)	(−4.6157)
*Lev*	−2.8557***	0.0260**	0.0143**	−0.5608***	0.0269**	0.0121*
	(−9.7227)	(2.0073)	(1.9736)	(−6.1010)	(2.0792)	(1.7745)
*Roa*	−3.0099***	−0.0907***	0.0167	0.6297***	−0.0856***	0.0011
	(−4.9530)	(−3.3890)	(1.0647)	(3.3109)	(−3.1984)	(0.0778)
*Cash*	0.5267	0.0222	−0.0261***	0.0305	0.0218	0.0081
	(1.3356)	(1.2788)	(−2.6066)	(0.2468)	(1.2552)	(0.8815)
*Aturn*	−1.7262***	−0.0427***	−0.0320***	0.2354***	−0.0401***	−0.0067**
	(−14.4692)	(−8.0544)	(−11.7460)	(6.3058)	(−7.6355)	(−2.3992)
*Top1*	−0.0159***	0.0005***	0.0006***	−0.0017*	0.0005***	0.0004***
	(−5.2831)	(3.4112)	(7.6748)	(−1.7657)	(3.4897)	(5.8833)
*Indep*	0.0280	–0.0047	0.0058***	0.0476**	–0.0046	0.0006
	(0.3605)	(−1.3767)	(2.8814)	(1.9607)	(−1.3361)	(0.3289)
*Dual*	0.2260**	−0.0083*	–0.0037	0.0601**	−0.0083*	−0.0038*
	(2.3304)	(−1.9365)	(−1.4538)	(1.9800)	(−1.9399)	(−1.6796)
*Soe*	−0.2938***	0.0134***	0.0213***	0.0191	0.0138***	0.0022
	(−2.6373)	(2.7407)	(7.5500)	(0.5467)	(2.8143)	(0.8389)
*Market*	0.7469***	0.0085	0.0020	–0.0240	0.0077	0.0058
	(4.5244)	(1.1685)	(0.4628)	(−0.4642)	(1.0567)	(1.5031)
*AGDP*	0.0004	−0.0019**	–0.0007	–0.0011	−0.0020**	–0.0006
	(0.0169)	(−1.9615)	(−1.2084)	(−0.1574)	(−1.9659)	(−1.2265)
*Year*	Yes	Yes	Yes	Yes	Yes	Yes
*Industry*	Yes	Yes	Yes	Yes	Yes	Yes
*City*	Yes	Yes	Yes	Yes	Yes	Yes
_cons	−4.1213**	1.1586***	0.0946*	−9.7629***	1.1287***	0.1427***
	(−1.9632)	(12.5417)	(1.7662)	(−14.8595)	(12.1029)	(2.9027)
Adj. *R*^2^	0.3566	0.2701	0.1050	0.4950	0.2702	0.2888
*N*	11560	11560	11560	11560	11560	11560

#### Reduce operating costs

The results in column (1) of Table 8 show that digital transformation significantly reduces the operating costs of enterprises, and columns (2)–(3) of Table 9 show the results of the mediating effect test for the mechanism of reducing operating costs. From the regression results, the regression coefficients of operating costs (Cost) are all significantly positive at the 1% level, and after controlling operating costs (Cost), the digital transformation (L. DIGI) coefficients are significantly negative, indicating that the impact of digital transformation on customer concentration is partially achieved by reducing operating costs. As mentioned earlier, the application of digital technologies can effectively streamline the internal operational processes of enterprises, integrate their internal operations, innovation activities and manufacturing processes, and realize real-time transmission and sharing of digital resources, thus effectively reducing internal management costs and product production costs. Secondly, the development of digital economy and the application of digital technology can reduce the information asymmetry in the upstream and downstream parts of the supply chain (Matt et al., 2015), increase the accuracy of the matching of supply and demand, effectively overcome the long inventory cycle of upstream enterprises and the shortage of goods in downstream enterprises, help enterprises dispatch upstream supplies in a more timely manner, greatly reduce the sales expenses and search costs, and achieve cost reduction and efficiency. Therefore, by reducing operating costs, enterprises can invest more resources in activities that can create greater value, and provide good technical support to improve customer satisfaction and reduce customer dependence.

**TABLE 8:** Heterogeneity test of regional digitalization level.

	(1)	(2)	(3)	(4)
	
	High level of regional digitization	Low level of regional digitization	High level of regional digitization	Low level of regional digitization
	*Cus_Top5*	*Cus_Top5*	*Cus_HHI*	*Cus_HHI*
*L.DIGI*	−0.0129***	–0.0009	−0.0045***	–0.0015
	(−5.6215)	(−1.5081)	(−3.8512)	(−1.2893)
*Size*	−0.0311***	−0.0360***	−0.0059***	−0.0072***
	(−10.8788)	(−10.4443)	(−4.0723)	(−4.5445)
*Lev*	0.0025	0.0398**	0.0063	0.0188*
	(0.1315)	(2.5392)	(0.6450)	(1.9546)
*Roa*	−0.1631***	0.0601***	–0.0291	0.0317
	(−4.1600)	(2.6559)	(−1.4642)	(1.5926)
*Cash*	0.0065	0.0577***	0.0127	0.0033
	(0.2652)	(3.1284)	(1.0247)	(0.2415)
*Aturn*	−0.0267***	−0.0318***	0.0003	−0.0143***
	(−3.4242)	(−4.3274)	(0.0793)	(−3.6959)
*Top1*	0.0004*	0.0003	0.0004***	0.0005***
	(1.9247)	(1.1545)	(3.8796)	(4.5741)
*Indep*	−0.0089*	–0.0065	0.0012	0.0005
	(−1.7821)	(−1.4850)	(0.4778)	(0.1997)
*Dual*	−0.0125**	–0.0040	–0.0036	–0.0049
	(−1.9908)	(−0.8290)	(−1.1384)	(−1.5143)
*Soe*	0.0127*	0.0301**	0.0013	0.0028
	(1.7270)	(2.1737)	(0.3433)	(0.7615)
*Market*	0.0062	0.0095*	0.0056	0.0115**
	(0.5172)	(1.6687)	(0.9083)	(2.1528)
*AGDP*	–0.0019	–0.0014	–0.0005	–0.0000
	(−1.4794)	(−0.9756)	(−0.7132)	(−0.0334)
*Year*	Yes	Yes	Yes	Yes
*Industry*	Yes	Yes	Yes	Yes
*City*	Yes	Yes	Yes	Yes
_cons	1.1922***	1.1882***	0.1059	0.1152*
	(4.6829)	(6.1371)	(0.8195)	(1.8879)
*R2*	0.2443	0.2336	0.2343	0.3898
*N*	5794	5766	5794	5766
*Chowtest*	χ^2^ = *4.56, P = 0.0000*		χ^2^ = *2.97, P = 0.0000*	

**TABLE 9:** Test results of mediating effects of operating costs and customer satisfaction.

	(1)	(2)	(3)	(4)	(5)	(6)
	*Cost*	*Cus_Top5*	*Cus_HHI*	*Market*	*Cus_Top5*	*Cus_HHI*
*L.DIGI*	−0.0058***	−0.0076***	−0.0026***	0.0012*	−0.0092***	−0.0029***
	(−4.5477)	(−4.8512)	(−3.1256)	(1.7489)	(−5.8894)	(−3.4689)
*Cost*		0.1657***	0.0455***			
		(14.4032)	(7.4491)			
*Market*					−0.0389*	−0.0283**
					(−1.8697)	(−2.5653)
*Size*	−0.0202***	−0.0291***	−0.0059***	0.0339***	−0.0309***	−0.0054***
	(−12.3547)	(−14.4803)	(−5.5299)	(37.9669)	(−14.6327)	(−4.8302)
*Lev*	0.1403***	0.0056	0.0075	−0.0231***	0.0207	0.0064
	(13.3916)	(0.4348)	(1.0887)	(−4.0190)	(1.6178)	(0.9436)
*Roa*	−0.8985***	0.0611**	0.0400***	−0.0578***	−0.0926***	0.0024
	(−41.4358)	(2.1479)	(2.6494)	(−4.7284)	(−3.3932)	(0.1665)
*Cash*	−0.1193***	0.0414**	0.0134	0.0170**	0.0198	0.0075
	(−8.4806)	(2.4019)	(1.4624)	(2.1800)	(1.1330)	(0.8094)
*Aturn*	0.0704***	−0.0526***	−0.0106***	0.0353***	−0.0441***	−0.0088***
	(16.5283)	(−9.9941)	(−3.7848)	(15.6189)	(−8.6568)	(−3.2443)
*Top1*	−0.0005***	0.0005***	0.0004***	0.0004***	0.0006***	0.0004***
	(−4.4323)	(4.1623)	(6.2727)	(7.0253)	(4.1896)	(6.0394)
*Indep*	0.0013	–0.0049	0.0004	0.0049***	−0.0059*	–0.0010
	(0.4629)	(−1.4599)	(0.2148)	(3.2159)	(−1.7356)	(−0.5707)
*Dual*	−0.0108***	–0.0067	–0.0035	0.0025	−0.0110**	−0.0049**
	(−3.1305)	(−1.5819)	(−1.5455)	(1.2786)	(−2.5523)	(−2.1307)
*Soe*	0.0367***	0.0077	0.0004	–0.0022	0.0151***	0.0049*
	(9.2211)	(1.5698)	(0.1702)	(−1.0221)	(3.2122)	(1.9563)
*Market*	0.0045	0.0070	0.0056	0.0017***	0.0002	−0.0012***
	(0.7722)	(0.9730)	(1.4708)	(4.4633)	(0.2129)	(−2.6477)
*AGDP*	0.0002	−0.0020**	–0.0006	0.0002	0.0004	0.0005
	(0.2543)	(−2.0138)	(−1.2396)	(0.3725)	(0.4482)	(0.9261)
*Year*	Yes	Yes	Yes	Yes	Yes	Yes
*Industry*	Yes	Yes	Yes	Yes	Yes	Yes
*City*	Yes	Yes	Yes	Yes	Yes	Yes
_cons	1.3553***	0.9382***	0.1113**	−0.7103***	0.9283***	0.1374***
	(18.0932)	(10.1020)	(2.2580)	(−36.3393)	(20.1582)	(5.6236)
Adj. *R*^2^	0.3365	0.2829	0.2911	0.2847	0.2017	0.2113
*N*	11560	11560	11560	11560	11560	11560

#### Customer satisfaction

This manuscript examines the customer satisfaction mechanism of digital transformation affecting customer concentration. Relative market share is used as a proxy for customer satisfaction because it laterally reflects the market share of a firm’s products or services, and a larger market share reflects customer recognition and satisfaction with a firm’s products or services (Kohtamäki et al., 2020). According to the empirical results in column (4), digital transformation does increase the relative market share of a firm, and columns (5)–(6) of Table 9 show the results of the mediating effect test for the mechanism of enhancing customer satisfaction. From the regression results, the regression coefficients of the relative market share of a firm (Market) are all significantly negative at least at the 10% level, after controlling the relative market share (Market). Digital transformation (L.DIGI) coefficients are significantly negative, indicating that the impact of digital transformation on customer concentration is partially achieved through the mechanism of increasing customer satisfaction. The above results suggest that digital transformation can increase a firm’s market share and effectively meet the needs of diverse customers, which in turn enhances customer satisfaction, improves customer structure diversification, and reduces customer concentration.

#### Heterogeneity analysis

This section examines the heterogeneous impact of digital transformation on different firms in terms of the internal and external environments. Specifically, the internal environment mainly includes the nature of property rights, the degree of market competition and the extent of customer interaction, and the external environment mainly refers to the regional digital environment.

#### The nature of corporate property rights

The impact of digital transformation on customer concentration varies with the nature of property rights. On the one hand, since SOEs generally bear the policy burden of maintaining social stability and securing employment (Kohtamäki et al., 2020), this policy bond gives SOEs a natural advantage in terms of market share, financing and lending, and other resource acquisition. As a result, SOEs face lower competition pressure and relatively pay less attention to innovative activities and cutting-edge digital technologies. In contrast, non-SOEs lack the support of relevant policy resources and face greater pressure to compete in the market. In order to improve their market competitiveness, non-state enterprises have a strong intention for innovation activities and the applications of cutting-edge digital technologies. Based on this, the samples are divided into state-owned enterprises and non-state-owned enterprises according to the nature of their property rights for group testing. The results are detailed in columns (1)–(4) of Table 10. Columns (1) and (3) show the regression results for the state-owned enterprises, and the coefficients of digital transformation (L.DIGI) fail to pass the significance test. Columns (2) and (4) show the regression results for the non-state-owned enterprises, and the coefficients of digital transformation (L.DIGI) are all significantly negative at the 1% level, indicating that the effect of digital transformation on the reduction of corporate customer concentration only exists among non-SOEs. It may be due to the stronger intention of non-SOEs to promote digital transformation in order to increase market share and gain a stronger competitive advantage in the market, which in turn reduces the dependence on major customers and promotes customer structure diversification.

**TABLE 10:** Heterogeneity test of the nature of corporate property rights.

	(1)	(2)	(3)	(4)
	State-owned enterprises	Non-state-owned enterprises	State-owned enterprises	Non-state-owned enterprises
	*Cus_Top5*	*Cus_Top5*	*Cus_HHI*	*Cus_HHI*
L.DIGI	–0.0045	−0.0104***	–0.0028	−0.0036***
	(−1.4243)	(−5.7566)	(−1.3446)	(−4.5548)
*Size*	−0.0366***	−0.0329***	−0.0112***	−0.0056***
	(−10.7521)	(−12.5199)	(−4.9919)	(−4.9110)
*Lev*	0.0277	0.0134	0.0384**	–0.0042
	(1.1814)	(0.8715)	(2.5022)	(−0.6195)
*Roa*	–0.0470	−0.0958***	0.0582	−0.0287**
	(−0.8600)	(−3.2219)	(1.6203)	(−2.2067)
*Cash*	0.0358	0.0080	–0.0035	0.0177**
	(1.0131)	(0.4083)	(−0.1507)	(2.0652)
*Aturn*	−0.0239***	−0.0472***	–0.0027	−0.0068**
	(−2.6705)	(−7.1337)	(−0.4567)	(−2.3624)
*Top1*	0.0004*	0.0001	0.0007***	0.0000
	(1.7056)	(0.4796)	(4.2438)	(0.3771)
*Indep*	0.0038	−0.0091**	0.0015	–0.0004
	(0.7172)	(−2.0277)	(0.4280)	(−0.1831)
*Dual*	–0.0178	–0.0051	−0.0162**	–0.0018
	(−1.5837)	(−1.1271)	(−2.1912)	(−0.8891)
*Market*	–0.0005	0.0138	0.0046	0.0073*
	(−0.0412)	(1.5307)	(0.5952)	(1.8688)
*AGDP*	0.0001	−0.0028**	0.0004	−0.0012**
	(0.0777)	(−2.4506)	(0.3720)	(−2.4878)
*Year*	Yes	Yes	Yes	Yes
*Industry*	Yes	Yes	Yes	Yes
*City*	Yes	Yes	Yes	Yes
_cons	1.0115***	1.1474***	0.1779	0.1620***
	(4.6126)	(11.0953)	(1.2362)	(3.5873)
adj. *R*^2^	0.4313	0.2457	0.4394	0.1559
*N*	3808	7752	3808	7752
*Chowtest*	χ^2^ = *6.78, P = 0.0000*		χ^2^ = *8.24, P = 0.0000*	

#### Customer interaction

Companies strengthen communication with customers by establishing product quality management systems, strategic mechanisms for sharing with business partners and integrity operation concepts, which help improve the customer participatory collaborative innovation model, strengthen customer monitoring and feedback channels and improve customer satisfaction. Therefore, this manuscript expects that customer interaction will enhance the effect of digital transformation on reducing customer concentration. Specifically, according to the information from the ESG (environmental, social, and governance aspects) of enterprises, this manuscript refers to the definitions of indicators in the Corporate Environmental, Social, and Governance (CESG) database, and complies data related to customer interaction, including three types of behaviors: whether enterprises have constructed product quality systems (C_qual), established strategic mechanisms and platforms for sharing with business partners (C_share, including long-term strategic cooperation agreements, shared experimental base, shared database, stable communication platforms, etc.), and established the concept of integrity management (C_inte, including integrity management, fair competition and institutional guarantee). This manuscript carries out group tests according to the above three types of behaviors.

According to the empirical results in Table 11, for the companies that have launched positive interactions with customers as shown in columns (1), (3), and (5), the coefficients of digital transformation (L.DIGI) are all significantly negative at least at the 10% level; columns (2), (4), and (6) refer to those that have not established interactions with customers related to product quality systems, strategy sharing mechanisms and platforms, and integrity management philosophy. None of the coefficients of digital transformation (L.DIGI) passed the significance test, and the results of Chowtest test showed significant differences between groups of group regressions on the presence or absence of product quality systems, strategic sharing mechanisms and platforms and honest management philosophy. The above results indicate the greater role of digital transformation in reducing customer concentration when there is positive interaction between enterprises and customers.

**TABLE 11:** Heterogeneity test of interaction with customers.^1^

	(1)	(2)	(3)	(4)	(5)	(6)
	Product quality system		Strategy sharing mechanism and platform		Integrity management	
	Yes	No	Yes	No	Yes	No
	*Cus_Top5*	*Cus_Top5*	*Cus_Top5*	*Cus_Top5*	*Cus_Top5*	*Cus_Top5*
*L.DIGI*	−0.0094**	–0.0007	−0.0081*	–0.0027	−0.0080**	–0.0003
	(−2.2921)	(−0.1313)	(−1.7701)	(−0.5552)	(−2.2813)	(−0.0298)
*Size*	−0.0228***	−0.0256***	−0.0265***	–0.0107	−0.0182***	−0.0349***
	(−4.7260)	(−3.5568)	(−5.2457)	(−1.5533)	(−4.6087)	(−2.7615)
Lev	0.0525	−0.0993*	0.0666	−0.0930**	–0.0438	–0.0571
	(1.4763)	(−1.8847)	(1.6060)	(−2.0993)	(−1.4361)	(−0.6547)
Roa	−0.1951***	0.0302	–0.0032	−0.2010**	−0.1787***	–0.1438
	(−2.6565)	(0.2889)	(−0.0342)	(−2.4856)	(−2.6879)	(−1.0230)
Cash	0.1291***	–0.1073	0.1046*	0.0003	0.0193	0.0088
	(2.6050)	(−1.5630)	(1.8258)	(0.0049)	(0.4571)	(0.0868)
Aturn	–0.0164	−0.1078***	–0.0159	−0.0621***	−0.0417***	−0.0736**
	(−1.2047)	(−5.3537)	(−1.0582)	(−3.6171)	(−3.9166)	(−2.1275)
Top1	–0.0005	0.0013***	–0.0005	0.0011***	0.0007***	0.0004
	(−1.3877)	(2.7089)	(−1.3561)	(2.6367)	(2.6183)	(0.4733)
Indep	–0.0033	–0.0046	0.0047	–0.0097	0.0012	−0.0329**
	(−0.4723)	(−0.4747)	(0.6008)	(−1.1494)	(0.2028)	(−2.0223)
Dual	0.0063	−0.0373**	−0.0330**	–0.0045	−0.0184*	–0.0080
	(0.5260)	(−2.1550)	(−2.3500)	(−0.3180)	(−1.7392)	(−0.3006)
Soe	0.0575***	0.0495***	0.0276**	0.0594***	0.0566***	0.0004
	(4.8791)	(3.0462)	(2.1277)	(4.3668)	(6.1593)	(0.0167)
Market	–0.0112	0.0364	0.0224	–0.0162	0.0028	–0.0189
	(−0.6377)	(1.4069)	(0.9972)	(−0.8100)	(1.4799)	(−0.5274)
AGDP	–0.0009	–0.0013	–0.0008	–0.0068	0.0009	0.0028
	(−0.3947)	(−0.4481)	(−0.3982)	(−1.5398)	(0.4724)	(0.6543)
Year	Yes	Yes	Yes	Yes	Yes	Yes
Industry	Yes	Yes	Yes	Yes	Yes	Yes
City	Yes	Yes	Yes	Yes	Yes	Yes
_cons	0.8953***	0.9802***	0.9673***	0.3957	0.5778***	1.2896**
	(3.1560)	(3.8271)	(4.5216)	(1.5008)	(6.6257)	(2.3150)
Adj. R^2^	0.3274	0.4509	0.3205	0.4029	0.2458	0.4581
N	1658	1082	1344	1396	2278	462
Chowtest	χ^2^ = 1.93, P = 0.0000		χ^2^ = 1.28, P = 0.0147		χ^2^ = 1.43, P = 0.0445	

^1^Given that corporate ESG reports are selective disclosures for companies, it reduces the number of observations in Table 8. Given the space, the regression results of customer concentration Cus_HHI are consistent with those of Cus_Top5, and the regression results of customer concentration Cus_HHI are left to be found.

#### The level of regional digitalization

The decision of digital transformation by enterprises depends on not only their own development needs but also the influence of the external environment (Kohtamäki et al., 2020). For enterprises, digital transformation is a systemic project with high risk and complexity, depending on the completeness of regional digital infrastructure. When a company is located in a region with well-developed digital infrastructure, the costs of digital transformation can be reduced and better external conditions for the success of digital transformation are provided. This manuscript argues that the higher level of regional digitization would bring a greater effect of digital transformation on the reduction of customer concentration. In this manuscript, four indicators including the Internet penetration rate, related practitioners, related output and cell phone penetration rate, are selected from the China Urban Statistical Yearbook. The comprehensive development index of digital economy is obtained by principal component analysis to construct indicators for the regional digitalization level. Table 8 reports the results of the heterogeneity test in terms of the regional digitalization level. According to the regression results, the regression coefficients of digital transformation (L.DIGI) in columns (1) and (3) are both significantly negative at the 1% level when the regional digitalization level is high; the regression coefficients of digital transformation (L.DIGI) in columns (2) and (4) fail to pass the significance test when the regional digitalization level is low. The above results indicate that the development of regional digital economy contributes to the success of corporate digital transformation, which in turn reduces the dependence on major customers.

## Further discussion

### Customer structure diversification

Generally speaking, companies diversify their customers by reducing customer concentration in two ways. One, by increasing the number of customers with whom a firm deals, which the firm makes a reality by conducting digital transformation, but this strategy cannot be verified because only the sales share of the top five customers is disclosed in the firm’s annual report. Second, increasing the diversification of the revenue streams of the firm can be done by decomposing the revenue share of the firm’s major customers according to the DuPont analysis and thus validating this strategy. Referring to Leung and Sun (2021), in order to observe more deeply the impact of digital transformation performed by firms on the diversification of their customer base. In this manuscript, the total sales of major customers with a sales share greater than 10% are decomposed into two parts using the DuPont analysis, as detailed in model (5).


(5) $$\frac{S\,a\,l\,e_{C\,C\,i,t}}{S\,a\,l\,e_{i,t}}=\frac{S\,a\,l\,e_{C\,C\,i,t}}{C\,o\,u\,n\,t_{i,t}}\times \frac{C\,o\,u\,n\,t_{i,t}}{S\,a\,l\,e_{i,t}}$$

where *Sale*_*CC*_ is a firm’s total sales to its major customers; *Sale* is a firm’s total sales in a given year. *Count* is the number of major customers a firm has in a given year, drawing on Leung and Sun (2021), which defines a single customer with a sales share of 10% or more as a firm’s major customer. *Sale_*CC*_/Count* is the number of sales per major Sales per customer; *Count/Sale* is the number of major customers per million sales. We perform a natural logarithm transformation on the two decomposed components. Table 12 shows that digital transformation does not significantly reduce supply chain sales per major customer, but significantly reduces the number of major customers per million sales. This indicates that the digital transformation of the company does not terminate the existing relationship with the major customers and it can be determined that the company has acquired new non-major customers and increased customer mix diversification.

**TABLE 12:** Regression results for the decomposition of customer structural diversity.^1^

	(1)	(2)
	Ln (1 + Sale_cci,t_/Count_i,t_)	Ln (1 + Count_i,t_ /Sale_i,t_)
L.DIGI	–0.0022	−0.0161***
	(−0.3202)	(−5.7295)
Size	1.0048***	−0.1132***
	(105.8377)	(−29.6183)
Lev	0.1807***	–0.0182
	(3.1517)	(−0.7902)
Roa	0.5609***	−0.2909***
	(5.1346)	(−6.6118)
Cash	0.2782***	0.0108
	(3.6020)	(0.3480)
Aturn	1.5175***	−0.1924***
	(58.6366)	(−18.4573)
Top1	0.0039***	0.0005*
	(6.3104)	(1.9266)
Indep	0.0302*	–0.0022
	(1.9171)	(−0.3477)
Dual	–0.0280	0.0282***
	(−1.4292)	(3.5774)
Soe	–0.0054	0.0050
	(−0.2307)	(0.5297)
Market	0.0662**	–0.0033
	(2.0659)	(−0.2536)
AGDP	0.0010	–0.0023
	(0.2208)	(−1.2164)
Year	Yes	Yes
Industry	Yes	Yes
City	Yes	Yes
_cons	−3.9678***	2.7773***
	(−10.8143)	(18.7961)
adj. R^2^	0.8764	0.3961
N	5154	5154

^1^In view of the fact that the diversification of the customer structure is discussed, a sample of more than 10% of the sales of a single customer is used, so that some of the major customer data that has not yet reached more than 10% of sales are not included.

### Corporate performance

There is no unified conclusion on the impact of customer concentration on corporate performance, and this manuscript follows this topic to explore whether customer structure diversification has a significant impact on performance, including return on assets (ROA), in the context of digital transformation of companies. Table 13 presents the relevant empirical results, columns (1) present the regression coefficients of customer concentration (L.Cus_Top5), which are significantly negative at least at the 5% level, indicating that customer concentration does reduce firm performance, and columns (2) present the customer diversification (L.Cus_diver) regression coefficients is significantly positive at least at the 5% level, indicating that customer structure diversification does enhance firm performance. Further, this manuscript divides the sample into high and low digital transformation according to the median annual industry of digital transformation and groups them to test the impact of customer structure diversification on firm performance in digitally transformed firms. The regression results in columns (3) and (4), show that the regression coefficients of customer structure diversification (L.Cus_diver) are significant at the 5% level in the sample with high degree of digital transformation, and in the subgroup with low degree of digital transformation, customer structure diversification (L.Cus_diver) does not pass the significance test, and the Chowtest test shows a significant difference. These results indicate that digital transformation contributes to customer structure diversification, reduces customer concentration, and promotes the improvement of corporate performance.

**TABLE 13:** Regression results of digital transformation, customer concentration and firm performance.

	(1)	(2)	(3)	(4)

	Full sample		High degree of digital transformation	Low degree of digital transformation
			
	ROA	ROA	ROA	ROA
*L.Cus_Top5*	−0.0102***			
	(−2.5777)			
*L.Cus_diver*		0.0102***	0.0121**	0.0094
		(2.5777)	(2.2059)	(1.6205)
*Size*	0.0140***	0.0140***	0.0119***	0.0151***
	(16.4282)	(16.4282)	(10.0185)	(12.1544)
*Lev*	−0.1256***	−0.1256***	−0.1080***	−0.1363***
	(−25.1208)	(−25.1208)	(−15.3469)	(−18.8463)
*Cash*	0.0520***	0.0520***	0.0559***	0.0528***
	(6.6931)	(6.6931)	(5.3769)	(4.5364)
*Aturn*	0.0298***	0.0298***	0.0291***	0.0290***
	(13.3489)	(13.3489)	(9.0460)	(9.1526)
*Top1*	0.0002***	0.0002***	0.0003***	0.0002*
	(4.3936)	(4.3936)	(4.3577)	(1.9178)
*Indep*	0.0032**	0.0032**	0.0070***	0.0009
	(2.1844)	(2.1844)	(3.4849)	(0.4371)
*Dual*	–0.0007	–0.0007	–0.0038	0.0020
	(−0.4123)	(−0.4123)	(−1.6300)	(0.6875)
*Soe*	−0.0103***	−0.0103***	−0.0081***	−0.0108***
	(−5.0172)	(−5.0172)	(−2.7197)	(−3.7213)
*Market*	–0.0026	–0.0026	–0.0038	–0.0001
	(−0.6619)	(−0.6619)	(−0.6665)	(−0.0206)
*AGDP*	–0.0001	–0.0001	0.0001	–0.0002
	(−0.1541)	(−0.1541)	(0.2663)	(−0.3764)
*Year*	Yes	Yes	Yes	Yes
*Industry*	Yes	Yes	Yes	Yes
*City*	Yes	Yes	Yes	Yes
_cons	−0.2851***	−0.2950***	−0.2781***	−0.2742***
	(−6.4067)	(−6.6612)	(−4.0656)	(−4.8056)
adj. *R*^2^	0.1974	0.1974	0.1990	0.2425
*N*	8828	8828	4376	4452
*Chowtest*			χ^2^ = *2.52, P = 0.0036*	

## Discussion

### Theoretical implications

This study contributes to the related literature research in the following ways. First, this manuscript focuses on the impact of digital transformation on customer concentration at the micro-firm level and extends the existing research perspective on customer concentration by eliciting potential mechanisms from dynamic capability theory. Previous studies focused on the economic consequences of customer concentration (Cohen and Li, 2020; Dhaliwal et al., 2020; Guo et al., 2020; Wang et al., 2021), but paid little attention to the factors influencing customer concentration (Leung and Sun, 2021). Although the recent literature addresses the relationship between digital technologies and customer purchase behavior (Hopkins, 2022; Kliestik et al., 2022; Nica et al., 2022), it focuses on retail business. Scholars have not studied the impact of digital transformation on their customer concentration at the micro-firm level. The research in this manuscript emphasizes the important role of digital transformation in reshaping the enterprise-customer relationship, and the integration of digitalization and dynamic capability theory provides a new perspective for this manuscript to study corporate customer relationship management in a new development stage.

Second, this manuscript is one of the first studies to introduce digital transformation to customer relationship management. Previous literature focused on the economic consequences of digital transformation itself [including corporate performance, social responsibility, etc. (Ferreira et al., 2019; Verhoef et al., 2021; Meng and Zhang, 2022)]. However, the impact of digital transformation on supply chain enterprises is relatively neglected, because the production and operation of enterprises do not exist alone and are deeply influenced by the upstream and downstream enterprises in their supply chains. In contrast, the research in this manuscript shows that digital transformation of enterprises does help optimize their customer structure and promote customer structure diversification, supporting the hypothesis of dynamic capability theory.

Finally, this manuscript reveals the variability of the role of digital transformation depending on the nature of enterprise ownership and the level of regional digital development. The findings suggest that the role of digital transformation in reducing customer concentration is stronger in non-state-owned enterprises and those in regions with a higher level of digital development.

### Practical implications

This manuscript discusses how digital transformation and the internal and external environment of enterprises reshape the enterprise-customer relationship. It has important practical implications for policy formulation by business managers and government agencies.

First, for enterprises, the implementation of digital transformation strategies enhances corporate innovation, attracts more customers, and diversifies the customer structure. Enterprises should follow the trend of digital transformation and use digital technologies to empower the customer relationship management. In addition, the corporate governance system should be improved to facilitate positive interactions with customers, promote the coupling of corporate operations and digital business, and improve corporate performance.

Second, for governments, digital construction is an important driver for China to advance high-quality economic development. The government should actively promote policies for digital development to provide a better external environment for enterprises’ digital transformation. At the same time, it should further deepen the reform of state-owned enterprises, strengthen the supervision and guidance of these enterprises, and enhance the enthusiasm of such enterprises to invest in digital transformation. In addition, targeted policies should be introduced to encourage enterprises in regions with lower levels of digital development to participate in digitalization.

## Conclusion

Digital transformation has a profound impact on both the internal operations and the innovative aspects of production and sales models, and concerns the development of digital industrialization and industrial digitization in China at this stage. This transformation has revolutionized the way companies build relationships with their key customers, suppliers, and other stakeholders, and reshaped the traditional interaction model between companies and their customers; therefore, it is crucial to explore the impact of digital transformation on customer relationships. However, existing research exploring the impact of digital transformation has overlooked its role in corporate customer relationships. This manuscript explores the mechanisms and economic consequences of digital transformation on the concentration of corporate customers by integrating dynamic capability theory and organizational learning theory. The empirical results show that, overall, digital transformation significantly reduces corporate customer concentration. That is, digital transformation effectively reduces the dependence of enterprises on large customers. In terms of impact mechanisms, digital transformation reduces firms’ reliance on large customers through three mechanisms: improving corporate innovation capabilities, reducing firms’ operating costs, and improving customer satisfaction. In terms of heterogeneity, the impact of digital transformation on reducing the dependence of non-state enterprises on large customers is greater relative to that of state-owned enterprises; the implementation of digital transformation strategies is more helpful for enterprises that have active interactions with customers to reduce their customer concentration; and the effect of digital transformation on reducing customer concentration is greater for enterprises in regions with higher levels of digital development relative to those in regions with lower levels of digital development. The economic consequence test finds that digital transformation diversifies customer structure and improves corporate performance. This study is a useful experiment of digital transformation in developing economies, and provides insights into the current digital ecosystem for companies to optimize their customer structure and thus reduce the risk of customer concentration.

## Limitations and future research direction

The research in this manuscript may have several shortcomings. First, the samples for research refer to A-share non-financial listed companies, because the data on digital transformation and customer relationship of listed companies are available. However, due to the variability of customer concentration in different industries and non-listed companies, targeted research needs to be done in the future. Second, the indicators for digital transformation in this manuscript are mainly derived from the data about the digital-related word frequency in the annual reports of listed companies, but they fail to better portray the specific details of the degree of digital transformation of corporate internal production processes.

Future research directions are stated as follows. First, on the one hand, the relationship between digital transformation and customer concentration of listed companies in different industries will be compared and analyzed, and the differential effect of digital transformation can be observed; on the other hand, the data on digital transformation of non-listed companies may be obtained by means of questionnaires, and more generalized research conclusions will be made. Second, in the future, the measurement of the degree of digital transformation will be further improved by adopting the proportion of corporate digital assets to digital transformation investment so as to supplement the relevant research in this manuscript.

## Data availability statement

The raw data supporting the conclusions of this article will be made available by the authors, without undue reservation.

## Author contributions

LL and SA: methodology and writing—review and editing. LL: formal analysis, data curation, and writing—original draft preparation. SA: investigation. SA and XL: supervision. LL, SA, and XL: conceptualization, read, and agreed to the published version of the manuscript.
